# Synthesis and Anticonvulsant Evaluation of some New 6-(Substituted-phenyl)thiazolo[3,2-b][1,2,4]triazole Derivatives in Mice 

**Published:** 2014

**Authors:** Xian-Qing Deng, Ming-Xia Song, Guo-Hua Gong, Shi-Ben Wang, Zhe-Shan Quan

**Affiliations:** a *Medical College, Jinggangshan University, Ji'an, JiangXi, 343009, China.*; b *Institute of Medicinal Chemistry and pharmacology, Inner Mongolia University for Nationalities, Tongliao, China.*; c *College of Pharmacy, Yanbian University, Yanji, Jilin, 133002, China.*

**Keywords:** Synthesis, Anticonvulsant, Thiazolotriazole, Maximal electroshock, Pentylenetetrazole

## Abstract

Epilepsy is the most frequent nearological affiction and afflicts 1% about of the world’s population. Currently there is an urgent need for the development of novel anticonvulsants with higher levels of potency and lower levels of toxicity. In this paper, a series of new 6-(substituted-phenyl)thiazolo[3,2-*b*][1,2,4]triazole derivatives were synthesized and tested for their anticonvulsant activities using the maximal electroshock (MES) and subcutaneous pentylenetetrazole (PTZ) screens, which are the most widely employed seizure models for early identification of candidate anticonvulsants. Their neurotoxicity was determined applying the rotarod test. In these compounds, 6-(4-fluorophenyl)thiazolo[3,2-*b*][1,2,4]triazole (3c) showed selective protection against the MES seizures with an ED_50_ value of 49.1 mg/Kg and a TD_50_ value of 94.1 mg/Kg, which provided compound 3c a protective index (PI = TD_50_/ED_50_) of 1.9 in the MES test. 6-(4-Propoxyphenyl)thiazolo[3,2-*b*][1,2,4]triazole (5b) was found to be active in both models*, i.e*. MES test and PTZ test. In the PTZ screen, compound 5b gave an ED_50_ of 63.4 mg/Kg and a TD_50_ of 105.6 mg/Kg, resulting in a PI value of 1.7 which is higher than carbamazepine.

## Introduction

Epilepsy is one of the most common neurological disorders, which is characterized by excessive temporary neuronal discharge resulting in recurrent unprovoked seizures (1, 2). It has been reported that about 1% of the world’s population (about 50 million people worldwide) are suffering with this neurological disorder at any one time (2). In recent years, significant efforts have been invested in the development of novel therapeutics, resulting in the emergence of several novel drugs as promising anticonvulsant agents (3, 4). However, the currently available anticonvulsants are effective in reducing the severity and frequency of seizures in less than 70% of patients. Up to 30% of patients are poorly treated with the available anticonvulsants (5, 6). Moreover, their usage is often associated with numerous undesirable side-effects (7-12). High levels of toxicity and intolerance, and a lack of efficacy also represent further limitations of the current anticonvulsant agents. With all of this in mind, there is an urgent need for the development of novel antiepileptic drugs (AEDs) with higher levels of potency and lower levels of toxicity.

1,2,4-Triazoles represent a key structure motif in medicinal chemistry and have been reported to exhibit a broad spectrum of biological activities, behaving as antifungal (13), anti-inﬂammatory (14, 15), anticancer (16, 17), antimicrobial (18, 19), and anticonvulsant (20, 21). Moreover, the chemistry of 1,2,4-triazoles and their fused heterocyclic derivatives has received considerable attention owing to their synthetic and biological importance. For example, a triazolo-thiazole system may be viewed as a cyclic analog of thiosemicarbazide, the latter often displays antimicrobial (22), anticancer (23), and anticonvulsant activities (24-26). Based on these facts, a novel series of 6-(substituted-phenyl)thiazolo[3,2-*b*][1,2,4]triazole derivatives were designed with an intention to synergize the anticonvulsant activity of 1,2,4-triazole and thiazole moiety in this paper. A benzene ring with substituents was introduced to the thiazole ring to increase the hydrophobicity of the whole structure.

**Figure 1:**
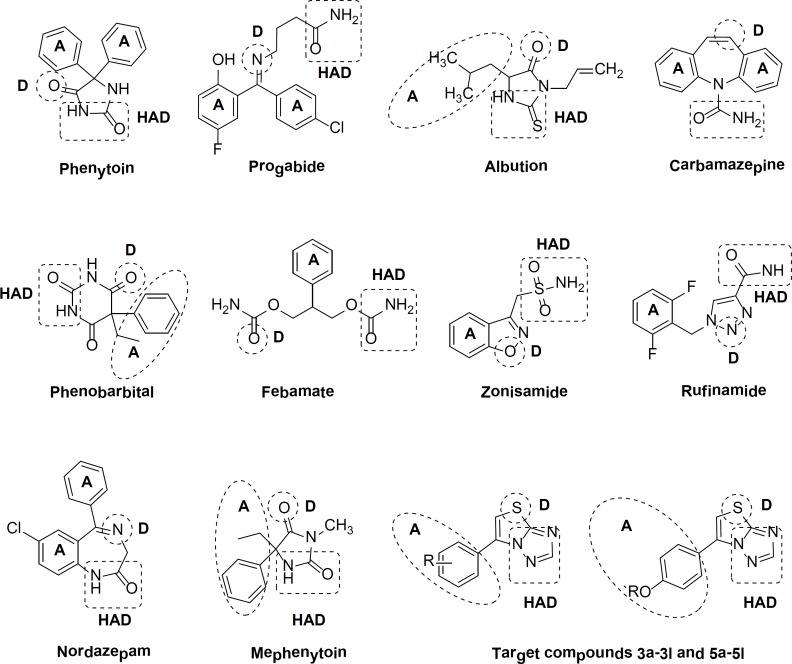
Pharmacophoric characteristics of well known antiepileptics and target compounds with the vital structural features: (A) hydrophobic unit, (D) electron donor group, hydrogen bond acceptor/donor unit (HAD).

These compounds designed contained a hydrophobic unit (R), an electron donor group (D), and a hydrogen donor/acceptor unit (HAD), which are the major characteristics associated with good anticonvulsant activity for the currently used anticonvulsant agents (as shown in Figure 1**)** (27). 

## Experimental

*The process of the synthesis and pharmacology*

**Scheme 1:**
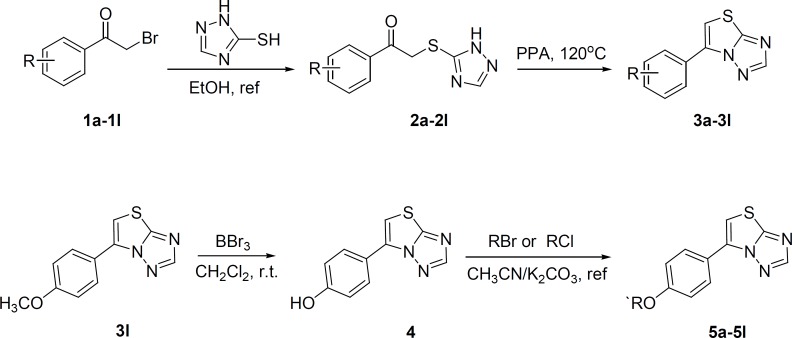
Synthetic route of target compounds (3a-3l, 5a-5l).

The target compounds (3a-3l and 5a-5l) were synthesized according to the route depicted in Scheme 1. Briefly, 6-phenylthiazolo[3,2-*b*][1,2,4]triazoles (3a-3l) were obtained by heating the 3-phenacylthio[1,2,4]triazoles (2a-2l) in the presence of polyphosphoric acid (28). The intermediates 2a-2l were easily prepared by the reaction of various phenacyl bromide with [1,2,4]triazole-3-thione in boiling ethanol. Compounds 5a-5l was achieved by reacting compound 4 with halogenated hydrocarbon in acetonitrile in the presence of K_2_CO_3_. The compound 4 was smoothly got by treating the compound 3l with boron tribromide. The structures of the desired compounds were confirmed by IR, ^1^H NMR, mass spectral and elemental analyses. The physicochemical properties of them are presented in the experimental section. Their anticonvulsant activities were all evaluated by maximal electroshock test (MES) and pentylenetetrazole (PTZ) model in mice, and their neurotoxicity were evaluated with the rotarod test.

*Materials and methods*

Melting points were determined in open capillary tubes and were uncorrected. IR spectra were recorded (in KBr) on a IRPrestige-21. 1H-NMR spectra were measured on an AV-300（Bruker, Fällanden, Switzerland）, and all chemical shifts were given in ppm relative to tetramethysilane. Mass spectra were measured on an HP1100LC (Agilent Technologies, Santa Clara, CA, USA). Elemental analyses were performed on a 204Q CHN (Perkin Elmer, Fremont, CA, USA). The major chemicals were purchased from Aldrich Chemical Corporation (Shanghai, China).

Preparation of compounds

*3-(substituted-phenacyl)thio[1,2,4]triazoles (* *2a* *-2l* *).*

[1,2,4]Triazole-3-thione (0.5 g, 0.005mol) was added to a solution of substituted-bromoacetophenone 1a-1l (0.77 g, 0.005 mol) in ethanol and the mixture was stirred under reflux for 4 hours. After cooling, the precipitated product was filtered and recrystallized from water to give 3-(substituted-phenacyl)thio[1,2,4]triazoles (2a-2l) in good yield.

*6-(substituted-phenyl)thiazolo[3,2-b][1,2,4]triazoles (* *3a* *-3l* *).*

3-(Substituted-phenacyl)thio[1,2,4]triazoles 2a-2l (0.04 mol) and polyphosphoric acid (8 g) were heated at 120 ^o^C for 2 hours. Then an aqueous solution of sodium hydrogen carbonate was added and the crude product was extracted twice by dichloromethane. The extracts were washed with water and dried over anhydrous MgSO_4_. After removing of the solvent under reduced pressure, the residue was crystallized from petroleum ether to give 6-(substituted-phenyl)thiazolo[3,2-*b*][1,2,4]triazoles *(3a-3l).*

*6-(4-hydroxy-phenyl)thiazolo[3,2-b][1,2,4]triazole* *(4)*

6-(4-Methoxy-phenyl)thiazolo[3,2-*b*][1,2,4]triazole (3l) (2.3 g, 0.01 mol) was dissolved in 50 mL dichloromethane. BBr_3_ (0.03 mol) was added dropwise to the solution and the mixture was stirred at room temperature. After 4 h the mixture was added slowly 20 mL ice cold water and allowed to stir for half hour. After removing the dichloromethane under reduced pressure, the resulting white precipitate was obtained by filtration.

*6-(4-(alkoxy)phenyl)thiazolo[3,2-b][1,2,4]triazole* *s (* *5a* *-5l* *)*.

K_2_CO_3_ (1.24 g, 0.009 mol) and 6-(4-hydroxy-phenyl)thiazolo[3,2-*b*][1,2,4]triazole 4 (0.003 mol) were dissolved in acetonitrile (50 mL) and refluxed for 30 min. Then alkyl bromide or benzyl chloride derivatives (0.0033 mol) were added into the mixture accompanied with some of benzyltriethylamine chloride (TEBA). The reaction mixture was stirred under reflux for 4-10 hours. After removing the solvent, 100 mL of water was added into the flask, which was extracted with dichloromethane (30 mL×3). The combined layer of dichloromethane was dried by anhydrous MgSO_4_. Evaporation of the solvent gave a crude product, which was purified by silica gel column chromatography with CH_2_Cl_2_-CH_3_OH (100:1) to a white solid. The yield, melting point and spectral data of each compound were given below.

*6-phenylthiazolo[3,2-b][1,2,4]triazole* *(* *3a* *)*

Mp 208-210 ^o^C, yield 36.8 %, Mol. weight: 201.25. ^1^H-NMR (CDCl_3_, 300 MHz): *δ* 7.14 (s, 1H, thiazole-H), 7.47-7.56 (m, 3H, Ar-H), 8.06-8.23 (m, 2H, Ar-H), 8.24 (s, 1H, triazole-H). IR (KBr) cm^-1^: 1634 1628 (C=N), 1175 (N-N). MS *m/z* 202 (M+1). *Anal*. Calcd. for C_10_H_7_N_3_S: C, 59.68; H, 3.51; N, 20.88. Found: C, 59.45; H, 3.42; N, 20.97.

*6-(2-fluorophenyl)thiazolo[3,2-b][1,2,4]triazole* *(3b)*

Mp 144-146^ o^C, yield 57.8 %, Mol. weight: 219.24. ^1^H-NMR (CDCl_3_, 300 MHz): δ 7.21 (s, 1H, thiazole-H), 7.27-7.46 (m, 3H, Ar-H), 8.23 (s, 1H, triazole-H), 8.53-8.59 (m, 4H, Ar-H). IR (KBr) cm^-1^: 1616 1608 (C=N), 1184 (N-N). MS *m/z *220 (M+1).* Anal*. Calcd. for C_10_H_6_FN_3_S: C, 54.78; H, 2.76; N, 19.17. Found: C, 54.57; H, 2.52; N, 19.31.

*6-(4-fluorophenyl)thiazolo[3,2-b][1,2,4]triazole (* *3c* *)*

Mp 150-152^ o^C, yield 55.2 %, Mol. weight: 219.24. ^1^H-NMR (CDCl_3_, 300 MHz): δ 7.10 (s, 1H, thiazole-H), 7.22 (d, 2H, *J* = 8.6 Hz, Ar-H), 8.09 (d, 2H, *J* = 8.6 Hz, Ar-H), 8.23 (s, 1H, triazole-H). IR (KBr) cm^-1^: 1624 1611 (C=N), 1181 (N-N). MS *m/z* 220 (M+1).* Anal*. Calcd. for C_10_H_6_FN_3_S: C, 54.78; H, 2.76; N, 19.17. Found: C, 54.54; H, 2.57; N, 19.34.

*6-(2-chlorophenyl)thiazolo[3,2-b][1,2,4]triazole (* *3d* *)*

Mp 100-102^ o^C, yield 62.2 %, Mol. weight: 235.69. ^1^H-NMR (CDCl_3_, 300 MHz): δ 7.28 (s, 1H, thiazole-H), 7.43-7.58 (m, 3H, Ar-H), 7.83-7.86 (m, 1H, Ar-H), 8.19 (s, 1H, triazole-H). IR (KBr) cm^-1^: 1634 1628 (C=N), 1175 (N-N). MS *m/z* 236 (M+1).* Anal*. Calcd. for C_10_H_6_ClN_3_S: C, 50.96; H, 2.57; N, 17.83. Found: C, 50.73; H, 2.39; N, 17.98.

*6-(3-chlorophenyl)thiazolo[3,2-b][1,2,4]triazole (* *3e* *)*

Mp 100-102^ o^C, yield 62.2 %, Mol. weight: 235.69. ^1^H-NMR (CDCl_3_, 300 MHz): δ 7.20 (s, 1H, thiazole-H), 7.45-7.48 (m, 2H, Ar-H), 8.01-8.24 (m, 2H, Ar-H), 8.25 (s, 1H, triazole-H). IR (KBr) cm^-1^: 1636 1619 (C=N), 1168 (N-N). MS *m/z* 236 (M+1).* Anal*. Calcd. for C_10_H_6_ClN_3_S: C, 50.96; H, 2.57; N, 17.83. Found: C, 50.71; H, 2.31; N, 18.10.

*6-(4-chlorophenyl)thiazolo[3,2-b][1,2,4]triazole (* *3f* *)*

Mp 100-102^ o^C, yield 62.2 %, Mol. weight: 235.69. ^1^H-NMR (CDCl_3_, 300 MHz): δ 7.16(s, 1H, thiazole-H), 7.49 (d, 2H, *J *= 8.6 Hz, Ar-H), 8.06 (d, 2H, *J *= 8.6 Hz, Ar-H), 8.24 (s, 1H,triazole-H). IR (KBr) cm^-1^: 1640 1624 (C=N), 1171 (N-N). MS *m/z* 236 (M+1).* Anal*. Calcd. for C_10_H_6_ClN_3_S: C, 50.96; H, 2.57; N, 17.83. Found: C, 50.74; H, 2.35; N, 18.04.

*6-(2-bromophenyl)thiazolo[3,2-b][1,2,4]triazole* *(* *3g* *)*

Mp 148-150^ o^C, yield 49.4 %, Mol. weight: 280.14. ^1^H-NMR (CDCl_3_, 300 MHz): δ 7.22 (s, 1H, thiazole-H), 7.37-7.48 (m, 2H, Ar-H), 7.70-7.77 (m, 2H, Ar-H), 8.17 (s, 1H, triazole-H). IR (KBr) cm^-1^: 1634 1564 (C=N), 1179 (N-N). MS *m/z* 280 (M^+^).* Anal*. Calcd. for C_10_H_6_BrN_3_S: C, 42.87; H, 2.16; N, 15.00. Found: C, 43.13; H, 2.02; N, 15.26.

*6-(3-bromophenyl)thiazolo[3,2-b][1,2,4]triazole (* *3h)*

Mp 156-158^ o^C, yield 64.6 %, Mol. weight: 280.14.^ 1^H-NMR (CDCl_3_, 300 MHz): δ 7.20 (s, 1H, thiazole -H), 7.37-7.62 (m, 2H, Ar-H), 8.05-8.26 (m, 2H, Ar-H), 8.25 (s, 1H, triazole-H). IR (KBr) cm^-1^: 1634 1570 (C=N), 1176 (N-N). MS *m/z* 280 (M^+^).* Anal*. Calcd. for C_10_H_6_BrN_3_S: C, 42.87; H, 2.16; N, 15.00. Found: C, 43.10; H, 2.07; N, 15.21.

*6-(4-bromophenyl)thiazolo[3,2-b][1,2,4]triazole (3i)*

Mp 184-186^ o^C, yield 60.4 %, Mol. weight: 280.14. ^1^H-NMR (CDCl_3_, 300 MHz): δ 7.17 (s, 1H thiazole-H), 7.65 (d, 2H, *J *= 8.6 Hz, Ar-H), 7.99 (d, 2H, *J *= 8.6 Hz, Ar-H), 8.23 (s, 1H, triazole-H). IR (KBr) cm^-1^: 1630 1583 (C=N), 1167 (N-N). MS *m/z* 280 (M^+^). *Anal*. Calcd. for C_10_H_6_BrN_3_S: C, 42.87; H, 2.16; N, 15.00. Found: C, 42.74; H, 2.22; N, 15.13.

*6-(4-nitrophenyl)thiazolo[3,2-b][1,2,4]triazole (* *3j* *)*

Mp 224-226^ o^C, yield 67.4 %, Mol. weight: 246.25. ^1^H-NMR (CDCl_3_, 300 MHz): δ 7.42 (s, 1H thiazole-H), 8.28 (s, 1H, triazole-H), 8.38 (s, 4H, Ar-H). IR (KBr) cm^-1^: 1642 1635 (C=N), 1180 (N-N). MS *m/z* 247 (M+1).* Anal*. Calcd. for C_10_H_6_N_4_O_2_S: C, 48.78; H, 2.46; N, 22.75. Found: C, 48.55; H, 2.61; N, 22.89.

*6-(4-methylphenyl)thiazolo[3,2-b][1,2,4]triazole* *(* *3k* *)*

Mp 176-178^ o^C, yield 42.3 %, Mol. Weight: 215.27.^ 1^H-NMR (CDCl_3_, 300 MHz): δ 2.42 (s, 3H, Ar-CH_3_), 7.08(s, 1H, thiazole-H), 7.32 (d, 2H, *J *= 8.1 Hz, Ar-H), 7.97 (d, 2H, *J *= 8.1 Hz, Ar-H), 8.22 (s, 1H, triazole-H). IR (KBr) cm^-1^: 1611 1578 (C=N), 1179 (N-N). MS *m/z *216 (M+1). *Anal*. Calcd. for C_11_H_9_N_3_S: C, 61.37; H, 4.21; N, 19.52. Found: C, 61.18; H, 4.34; N, 19.74.

*6-(4-methoxyphenyl)thiazolo[3,2-b][1,2,4]triazole (* *3l* *)*

Mp 178-180^ o^C, yield 45.2 %, Mol. Weight: 231.27. ^1^H-NMR (CDCl_3_, 300 MHz): δ 3.87 (s, 3H, Ar -OCH_3_), 7.00 (s, 1H, thiazole-H), 7.04 (d, 2H,* J *= 8.8 Hz, Ar-H), 8.02 (d, 2H,* J *= 8.8 Hz, Ar-H), 8.22 (s, 1H, triazole-H). IR (KBr) cm^-1^: 1607 1573 (C=N), 1182 (N-N). MS *m/z* 232 (M+1).* Anal*. Calcd. for C_11_H_9_N_3_OS: C, 57.13; H, 3.92; N, 18.17. Found: C, 57.35; H, 4.10; N, 18.33.

*6-(4-hydroxy-phenyl)thiazolo[3,2-b][1,2,4]triazole* *(* *4* *)*

Mp 232-234^ o^C, yield 79.8 %, Mol. Weight: 217.25. ^1^H-NMR (DMSO-*d*_6_, 300 MHz): δ 4.83 (s, 1H, Ar-OH), 6.99 (s, 1H, thiazole-H), 7.04 (d, 2H,* J *= 8.7 Hz, Ar-H), 7.94 (d, 2H,* J *= 8.7 Hz, Ar-H), 8.21 (s, 1H, triazole-H). IR (KBr) cm^-1^: 1614 1566 (C=N), 1173 (N-N). MS *m/z* 218 (M+1). *Anal*. Calcd. for C_10_H_7_N_3_OS: C, 55.29; H, 3.25; N, 19.34. Found: C, 55.04; H, 3.18; N, 19.61.

*6-(4-ethoxyphenyl)thiazolo[3,2-b][1,2,4]triazole (* *5a* *)*

Mp 122-124^ o^C, yield 87.4 %, Mol. Weight: 245.30. ^1^H-NMR (CDCl_3_, 300 MHz): δ 1.45 (t, 3H, *J *= 6.9 Hz, CH_3_), 4.10 (q, 2H, *J *= 6.9 Hz, OCH_2_ ), 7.00 (s, 1H, thiazole-H), 7.02 (d, 2H,* J *= 8.8 Hz, Ar-H), 8.01 (d, 2H,* J *= 8.8 Hz, Ar-H), 8.22 (s, 1H, triazole-H). IR (KBr) cm^-1^: 1606 1569 (C=N), 1181 (N-N). MS *m/z* 246 (M+1).* Anal*. Calcd. for C_12_H_11_N_3_OS: C, 58.76; H, 4.52; N, 17.13. Found: C, 58.98; H, 4.40; N, 17.01.

*6-(4-propoxyphenyl)thiazolo[3,2-b][1,2,4]triazole* *(* *5b* *)*

Mp 82-84^ o^C, yield 89.3 %, Mol. Weight: 259.33. ^1^H-NMR (CDCl_3_, 300 MHz): δ 1.06 (t, 3H, *J *= 7.3 Hz, CH_3_), 1.81-1.88 (m, 2H, CH_2_), 3.99 (q, 2H, *J *= 6.5 Hz, OCH_2_ ), 6.99 (s, 1H, thiazole-H), 7.02 (d, 2H,* J *= 8.5 Hz, Ar-H), 8.01 (d, 2H,* J *= 8.5 Hz, Ar-H), 8.22 (s, 1H, triazole-H). IR (KBr) cm^-1^: 1602 1569 (C=N), 1158 (N-N). MS *m/z* 260.3 (M+1). *Anal*. Calcd. for C_13_H_13_N_3_OS: C, 60.21; H, 5.05; N, 16.20. Found: C, 60.05; H, 5.16; N, 16.46.

*6-(4-butoxyphenyl)thiazolo[3,2-b][1,2,4]triazole (* *5c* *)*

Mp 102-104^ o^C, yield 87.1 %, Mol. Weight: 273.35. ^1^H-NMR (CDCl_3_, 300 MHz): δ 1.00 (t, 3H, *J *= 7.4 Hz, CH_3_), 1.51-1.81 (m, 4H, (CH_2_)_2_), 4.03 (q, 2H, *J *= 6.5 Hz, OCH_2_ ), 7.00 (s, 1H, thiazole-H), 7.03 (d, 2H,* J *= 8.9 Hz, Ar-H), 8.01 (d, 2H,* J *= 8.9 Hz, Ar-H), 8.23 (s, 1H, triazole-H). IR (KBr) cm^-1^: 1601 1571 (C=N), 1164 (N-N). MS *m/z* 274.1 (M+1).* Anal*. Calcd. for C_14_H_15_N_3_OS: C, 61.51; H, 5.53; N, 15.37. Found: C, 61.75; H, 5.44; N, 15.59.

*6-(4-(pentyloxy)phenyl)thiazolo[3,2-b][1,2,4]triazole* *(* *5d* *)*

Mp 86-88^ o^C, yield 92.3 %, Mol. Weight: 287.38. ^1^H-NMR (CDCl_3_, 300 MHz): δ 0.94 (t, 3H, *J *= 7.0 Hz, CH_3_), 1.41-1.49 (m, 4H, (CH_2_)_2_), 1.77-1.85 (m, 2H, CH_2_), 4.02 (q, 2H, *J *= 6.6 Hz, OCH_2_ ), 6.99 (s, 1H, thiazole-H), 7.02 (d, 2H,* J *= 8.9 Hz, Ar-H), 8.01 (d, 2H,* J *= 8.9 Hz, Ar-H), 8.22 (s, 1H, triazole-H). IR (KBr) cm^-1^: 1597 1564 (C=N), 1159 (N-N). MS *m/z* 288 (M+1).* Anal*. Calcd. for C_15_H_17_N_3_OS: C, 62.69; H, 5.96; N, 14.62. Found: C, 62.42; H, 6.12; N, 14.86.

*6-(4-(heptyloxy)phenyl)thiazolo[3,2-b][1,2,4]triazole* *(* *5e* *)*

Mp 76-78^ o^C, yield 90.7 %, Mol. Weight: 315.43. ^1^H-NMR (CDCl_3_, 300 MHz): δ 0.90 (t, 3H, *J *= 6.4 Hz, CH_3_), 1.33-1.48 (m, 8H, (CH_2_)_4_), 1.79-1.84 (m, 2H, CH_2_), 4.02 (q, 2H, *J *= 6.5 Hz, OCH_2_ ), 6.99 (s, 1H, thiazole-H), 7.02 (d, 2H,* J *= 8.7 Hz, Ar-H), 8.01 (d, 2H,* J *= 8.7 Hz, Ar-H), 8.22 (s, 1H, triazole-H). IR (KBr) cm^-1^: 1587 1553 (C=N), 1143 (N-N). MS *m/z* 316 (M+1).* Anal*. Calcd. for C_17_H_21_N_3_OS: C, 64.73; H, 6.71; N, 13.32. Found: C, 64.95; H, 6.63; N, 13.51.

*6-(4-(benzyloxy)phenyl)thiazolo[3,2-b][1,2,4]triazole* *(* *5f* *)*

Mp 123-124^ o^C, yield 79.6 %, Mol. Weight: 307.37. ^1^H-NMR (CDCl_3_, 300 MHz): δ 5.14 (s, 2H, OCH_2_ ), 7.00 (s, 1H, thiazole-H), 7.11 (d, 2H,* J *= 8.9 Hz, Ar-H), 7.37-7.47 (m, 5H, Ar-H), 8.02 (d, 2H,* J *= 8.9 Hz, Ar-H), 8.23 (s, 1H, triazole-H). IR (KBr) cm^-1^: 1609 1572 (C=N), 1181 (N-N). MS *m/z* 308 (M+1).* Anal*. Calcd. for C_17_H_13_N_3_OS: C, 66.43; H, 4.26; N, 13.67. Found: C, 66.20; H, 4.35; N, 13.89.

*6-(4-(2-fluorobenzyloxy)phenyl)thiazolo[3,2-b][1,2,4]triazole* *(* *5g* *)*

Mp 102-104^ o^C, yield 76.5 %, Mol. weight: 325.36. ^1^H-NMR (CDCl_3_, 300 MHz): δ 5.21 (s, 2H, OCH_2_ ), 7.01 (s, 1H, thiazole-H), 7.11-7.52 (m, 6H, Ar-H), 8.03 (d, 2H,* J *= 8.9 Hz, Ar-H), 8.22 (s, 1H, triazole-H). IR (KBr) cm^-1^: 1602 1580 (C=N), 1179 (N-N). MS *m/z* 326 (M+1).* Anal*. Calcd. for C_17_H_12_FN_3_OS: C, 62.76; H, 3.72; N, 12.91. Found: C, 62.94; H, 3.77; N, 12.82.

*6-(4-(3-fluorobenzyloxy)phenyl)thiazolo[3,2-b][1,2,4]triazole (5h)*

Mp 88-90^ o^C, yield 77.5 %, Mol. Weight: 325.36. ^1^H-NMR (CDCl_3_, 300 MHz): δ 5.13 (s, 2H, OCH_2_ ), 7.01 (s, 1H, thiazole-H), 7.06-7.37 (m, 6H, Ar-H), 8.03 (d, 2H,* J *= 8.9 Hz, Ar-H), 8.22 (s, 1H, triazole-H). IR (KBr) cm^-1^: 1598 1575 (C=N), 1172 (N-N). MS *m/z* 326.3 (M+1).* Anal*. Calcd. for C_17_H_12_FN_3_OS: C, 62.76; H, 3.72; N, 12.91. Found: C, 62.90; H, 3.81; N, 12.78.

*6-(4-(4-fluorobenzyloxy)phenyl)thiazolo[3,2-b][1,2,4]triazole (* *5i* *)*

Mp 140-142^ o^C, yield 84.1 %, Mol. Weight: 325.36. ^1^H-NMR (CDCl_3_, 300 MHz): δ 5.10 (s, 2H, OCH_2_ ), 7.01 (s, 1H, thiazole-H), 7.06-7.12 (m, 4H, Ar-H), 7.42 (d, 2H,* J *= 8.2 Hz, Ar-H), 8.03 (d, 2H,* J *= 8.9 Hz, Ar-H), 8.22 (s, 1H, triazole-H). IR (KBr) cm^-1^: 1605 1583 (C=N), 1182 (N-N). MS *m/z* 326.2 (M+1).* Anal*. Calcd. for C_17_H_12_FN_3_OS: C, 62.76; H, 3.72; N, 12.91. Found: C, 62.64; H, 3.63; N, 12.99.

*6-(4-(2-chlorobenzyloxy)phenyl)thiazolo[3,2-b][1,2,4]triazole* *(* *5j* *)*

Mp 98-100^ o^C, yield 80.9 %, Mol. Weight: 341.81. ^1^H-NMR (CDCl_3_, 300 MHz): δ 5.25 (s, 2H, OCH_2_ ), 7.01 (s, 1H, thiazole-H), 7.12 (d, 2H,* J *= 8.8 Hz, Ar-H), 7.26-7.57 (m, 4H, Ar-H), 8.04 (d, 2H,* J *= 8.8 Hz, Ar-H), 8.22 (s, 1H, triazole-H). IR (KBr) cm^-1^: 1594 1575 (C=N), 1171 (N-N). MS *m/z* 342.1 (M+1).* Anal*. Calcd. for C_17_H_12_ClN_3_OS: C, 59.73; H, 3.54; N, 12.29. Found: C, 59.61; H, 3.59; N, 12.47.

*6-(4-(3-chlorobenzyloxy)phenyl)thiazolo[3,2-b][1,2,4]triazole (* *5k* *)*

Mp 124-126^ o^C, yield 68.7%, Mol. Weight: 341.81. ^1^H-NMR (CDCl_3_, 300 MHz): δ 5.11 (s, 2H, OCH_2_ ), 7.01 (s, 1H, thiazole-H), 7.09 (d, 2H,* J *= 8.8 Hz, Ar-H), 7.32-7.46 (m, 4H, Ar-H), 8.04 (d, 2H,* J *= 8.8 Hz, Ar-H), 8.22 (s, 1H, triazole-H). IR (KBr) cm^-1^: 1590 1571 (C=N), 1167 (N-N). MS *m/z* 342 (M+1).* Anal*. Calcd. for C_17_H_12_ClN_3_OS: C, 59.73; H, 3.54; N, 12.29. Found: C, 59.56; H, 3.62; N, 12.50.

*6-(4-(4-chlorobenzyloxy)phenyl)thiazolo[3,2-b][1,2,4]triazole (* *5l* *)*

Mp 141-143^ o^C, yield 69.5 %, Mol. Weight: 341.81. ^1^H-NMR (CDCl_3_, 300 MHz): δ 5.10 (s, 2H, OCH_2_ ), 7.01 (s, 1H, thiazole-H), 7.09 (d, 2H,* J *= 8.6 Hz, Ar-H), 7.38 (s, 4H, Ar-H), 8.03 (d, 2H,* J *= 8.6 Hz, Ar-H), 8.22 (s, 1H, triazole-H). IR (KBr) cm^-1^: 1598 1582 (C=N), 1175 (N-N). MS *m/z* 342.3 (M+1).* Anal*. Calcd. for C_17_H_12_ClN_3_OS: C, 59.73; H, 3.54; N, 12.29. Found: C, 59.52; H, 3.42; N, 12.21.

*Pharmacology*

Mail KunMing mice (supplied by the Laboratory of Animal Research, Yanbian University, China) weighting 18–22 g were used for pharmacological study. Animals were allowed free access to food and water except during the experiment and housed at controlled room temperature with 12 h light/dark schedule. All compounds were dissolved in dimethyl sulfoxide (DMSO) with the injection volume of 0.05 mL per 20 g, which had no effect on the test system.

*Anticonvulsant effects in the MES test (* 29 *,* 30 *)*

The MES test was carried out using the methods described in the anticonvulsant drug development (ADD) program of the National Institutes of Health (USA). Seizures were elicited with a 60 Hz alternating current of 50 mA intensity in mice. The current was applied via corneal electrodes for 0.2 s. Protection against the spread of MES-induced seizures was defined as the abolition of the hind leg and tonic maximal extension component of the seizure. Animals were given intraperitoneal injection (*i.p*.) of the test compounds in the MES test. At 30 min after the administration of the compounds, the activities were evaluated in MES test. In phase-I screening, each compound was administered at the dose levels of 30, 100, and 300 mg/Kg for evaluating the preliminary anticonvulsant activity. For determination of the median effective dose (ED_50_) and the median toxic dose (TD_50_), the phase-II screening was prepared. Several groups (each group of 10 mice) were given various intraperitoneal doses of the tested compound until at least three points were established in the range of 10–90% seizure protection or neurotoxicity. The number of animals per group protected against MES (or neurotoxic in the rotarod test) is converted to a percentage, and a dose–response curve can be constructed. Then the respective ED_50_ and TD_50_ values, 95% confidence intervals were calculated by the statistics software SPSS 13.0 with probit analysis.

*Neurotoxicity (NT)* *screening (*29*,*^30^*)*

The neurotoxicity of the compounds was measured in mice by the rotarod test. The mice were trained to stay on a rotarod of diameter 3.2 cm that rotates at 10 rpm. Trained animals were given *i.p*. of the test compounds. Neurotoxicity was indicated by the inability of the animal to maintain equilibrium on the rod for at least 1min in each of the trials. 

*PTZ-induced seizures (* 29 *,* 30 *)*

The PTZ test utilizes a dose of pentylenetetrazole (85 mg/kg). PTZ can produce clonic seizures lasting for at least five seconds in 97 percent of animals tested. At 30 min after the administration (*i.p*.) of the test compound, 85 mg/Kg PTZ dissolved in saline was administered subcutaneouly. Animals are observed over a 30 minute period. Absence of clonic spasms in the observed time period indicates that the compound has the ability to abolish the effect of pentylenetetrazole on seizure threshold.

*Log P* *calculation *

The calculated Log P (miLog P) values were calculated using the Molinspiration online property calculation toolkit (31).

## Results and Discussion

*Anticonvulsant activity*

A very important step in antiepileptic drug discovery is the choice of an appropriate animal model for the initial screening. At present, there are three models *in-vivo* - the MES, the PTZ, and the kindling model - which are routinely used by most AEDs discovery programs. Of these, the MES and PTZ seizure models represent the two animal seizure models most widely used in the search for new AEDs (32, 33). The MES test is thought to predict drugs effective against generalized seizures of the tonic-clonic (grand mal) type, whereas the PTZ test is used to find drugs effective against the generalized seizures of the petit mal (absence) type. In this study, the two models were used for screening the anticonvulsant activity of target compounds. In the preliminary evaluation of anticonvulsant activities (Phase I), doses of 30, 100, and 300 mg/Kg were used in both models, and the results were presented in Table 1. 

**Table 1:** Phase I anticonvulsant screening of the compounds in mice^a^

**Comp.** **No.**	**R**	**MES** b			**PTZ** b			**NT ** b			**miLogP**
		30^b^	100	300	30	100	300	30	100	300	-
3a	H	-	1/3^c^	3/3	-	0/3	0/3	0/3	0/3	3/3	2.1
3b	2-F	-	1/3	2/3	-	0/3	0/3	0/3	1/3	3/3	2.2
3c	4-F	1/3	3/3	3/3	-	0/3	0/3	0/3	2/3	3/3	2.3
3d	2-Cl	-	0/3	1/3	-	0/3	0/3	0/3	0/3	1/3	2.8
3e	3-Cl	-	0/3	0/3	-	1/3	2/3	0/3	0/3	0/3	2.8
3f	4-Cl	-	0/3	0/3	-	0/3	1/3	0/3	0/3	0/3	2.8
3g	2-Br	-	0/3	0/3	-	0/3	0/3	0/3	0/3	0/3	2.9
3h	3-Br	-	0/3	1/3	-	0/3	2/3	0/3	0/3	1/3	2.9
3i	4-Br	-	0/3	3/3	0/3	2/3	3/3	0/3	1/3	3/3	2.9
3j	4-NO_2_	-	0/3	2/3	-	0/3	0/3	0/3	0/3	2/3	2.1
3k	4-CH_3_	-	1/3	3/3	-	0/3	0/3	0/3	0/3	2/3	2.6
3l	4-OCH_3_	-	1/3	3/3	-	1/3	2/3	0/3	1/3	3/3	2.2
5a	C_2_H_5_	-	0/3	1/3	-	0/3	0/3	0/3	0/3	1/3	2.6
5b	C_3_H_7_	0/3	2/3	3/3	1/3	3/3	3/3	0/3	2/3	3/3	3.1
5c	C_4_H_9_	-	1/3	3/3	-	1/3	2/3	0/3	2/3	3/3	3.6
5d	C_5_H_11_	-	0/3	2/3	-	0/3	0/3	0/3	1/3	3/3	4.1
5e	C_7_H_15_	-	0/3	0/3	-	0/3	0/3	0/3	0/3	0/3	5.1
5f	CH_2_C_6_H_5_	-	0/3	0/3	-	0/3	0/3	0/3	0/3	0/3	3.8
5g	CH_2_C_6_H_5_(2-F)	-	0/3	0/3	-	0/3	0/3	0/3	0/3	0/3	3.9
5h	CH_2_C_6_H_5_(3-F)	-	0/3	0/3	-	0/3	0/3	0/3	0/3	0/3	3.9
5i	CH_2_C_6_H_5_(4-F)	-	0/3	0/3	-	0/3	0/3	0/3	0/3	0/3	4.0
5j	CH_2_C_6_H_5_(2-Cl)	-	0/3	0/3	-	0/3	0/3	0/3	0/3	0/3	4.4
5k	CH_2_C_6_H_5_(3-Cl)	-	0/3	0/3	-	0/3	0/3	0/3	0/3	0/3	4.4
5l	CH_2_C_6_H_5_(4-Cl)	-	0/3	0/3	-	0/3	0/3	0/3	0/3	0/3	4.5

All positive reaction numbers are in bold italic.

a : Animals was administered intraperitoneal injection.

b : Doses of 30, 100 and 300 mg/Kg were administered in maximal electroshock seizure test (MES), pentylenetetrazole-induced seizure test (PTZ), and neurotoxicity (NT) tests.

c : The figures n/n indicate the number of animals protected/number of animals tested. The number of mice used is three. Sign “-” in the table means not tested.

According to the results of the anticonvulsant activity studies, 6-(4-fluorophenyl)thiazolo[3,2-*b*][1,2,4]triazole (3c) was highly selective and found to be the most active compound in MES test with the complete protection at the dose of 100 mg/Kg and partial protection (one-third) at the dose of 30 mg/Kg. In the same test, compound 3a, 3b, 3k, 3l, and 5c were protective at the dose of 100 mg/Kg with the proportion of one-third, compound 5b showed protection in two-thirds at the same dose. Compounds 3a-3d, 3h-3l, and 5a-5d showed activities against MES at 300 mg/Kg in varying degrees. 

In the PTZ model the most active compound of tested compounds was 6-(4-propoxyphenyl)thiazolo[3,2-*b*][1,2,4]triazole (5b), which showed the complete protection at the dose of 100 mg/Kg and partial protection (one-third) at the dose of 30 mg/Kg. Compound 3i, with a 4-bromo moiety, was found to have anticonvulsant activity at 100 mg/Kg dose with protection in two-thirds. While derivatives with 3-chloro (3e), 4-methoxyl (3l) and 4-butoxy (5c) substitution exhibited protection against PTZ in one-third at 100 mg/Kg dose. At the dose of 300 mg/Kg, 3e, 3f, 3h, 3i, 3l, and 5b-5c showed activities against PTZ in varying degrees. 

It is well accepted that blood-brain barrier (BBB) is an important selective barrier on the drug's way to the central nervous system. Overcoming the difficulty of delivering therapeutic agents to specific regions of the brain presents a major challenge to treatment of most brain disorders. According to Kaliszan *et al.* (34), lipophilicity (logP) and molecular weight (MW) of the compound are the main factors affecting drug delivery across the BBB. From the calculated LogP parameters of the prepared compounds (3a-3l, and 5a-5l), it can be observed that all compounds exhibited a nice LogP ranging from 2.1 to 5.1, which would enable the compounds to penetrate the BBB (Table 1). In group 5a-5l, however, only four compounds (5a-5d) with relatively small substituents showed anticonvulsant activities in MES or PTZ test, as the sizes of substituents increased, the anticonvulsant activity of them disappeared (5e-5l). This may be due to the big lipophilicity of the molecules, which interrupted the absorption and distribution of these compounds sequentially reduced bioavailability (35). The above considerations were also in agreement with the theory that there was an optimum Log P for the drugs acting on the central nervous system, and the drugs with this optimum Log P will be least inhibited in their movement through the aqueous and lipophilic phases of living tissue (36). Another contributor for the non-activity of compounds 5e-5l may be the steric hindrance formed by big size of the substitution, which may drop their affinity to some assumed target receptors.

From the rotarod test results, it seems that compounds, with anticonvulsant activities in MES/PTZ test, exhibited neurotoxicity at the same doses. For example, compounds 3c and 5b with high activity also displayed serious neurotoxicity.

**Table 2:** Phase II anticonvulsant evaluation in mice^a^.

**Compound**	**ED**_50_ **(MES)**^b^	**ED**_50_ **(PTZ)**^c^	**TD**_50_ d	**PI** **(TD**_50_**/ ED**_50_**) **	
				**MES**	**PTZ**
3c	49.1 (44.4-54.3)	-	94.7 (86.1-104.2)	1.9	-
5b	-e	63.4 (55.0-73.1)	105.6 (92.4-120.7)	-	1.7
Carbamazepine	9.8 (8.9-10.8)	>100	44.0 (40.2-48.1)	4.5	<0.44

a : All animals was administered intraperitoneal injection.

b : The median effective dose (ED_50_) was measured in maximal electroshock seizure test, confidence intervals given in the bracket and the unit is mg/Kg.

c : The median effective dose (ED_50_) was measured in pentylenetetrazole-induced seizure test, confidence intervals given in the bracket and the unit is mg/Kg.

d : The median neurotoxic dose (TD_50_) was measured in the rotarod test, confidence intervals given in the bracket and the unit is mg/Kg.

e : Not tested.

Compounds 3c and 5b were selected for quantification of the pharmacological parameters (ED_50_ and TD_50_). Results of the quantitative test for the compounds, along with the data of the standard drugs carbamazepine, are reported in Table 2. In the MES screen, 6-(4-fluorophenyl)thiazolo[3,2-*b*][1,2,4]triazole (3c) showed an ED_50_ and protective index (PI) value of 49.1 and 1.9. In the PTZ screen, 6-(4-propoxyphenyl)thiazolo[3,2-*b*][1,2,4]triazole 5b gave an ED_50_ of 63.4 mg/Kg and a TD_50_ of 105.6 mg/Kg, resulting in a high pi-value of 1.7 when compared to carbamazepine (PI < 0.44). 

## Conclusion

A series of new 6-(substituted-phenyl)thiazolo[3,2-*b*][1,2,4]triazole derivatives were synthesized and studied for their anticonvulsant activity using MES and PTZ tests. Among the compounds synthsized, two compounds (3c and 5b) were found to have promising anticonvulsant activities in the models employed for anticonvulsant evaluation. 6-(4-Fluorophenyl)thiazolo[3,2-*b*][1,2,4]triazole (3c) was highly selective and found to be the most active compound against MES seizures. 6-(4-Propoxyphenyl)thiazolo[3,2-*b*][1,2,4]triazole (5b) was active in both models. In the PTZ screen, compound 5b gave an ED_50_ of 63.4 mg/Kg and a TD_50_ of 105.6 mg/Kg, resulting in a high PI value of 1.7 when compared to carbamazepine (PI < 0.44). High Neurotoxicity is the main problem of this series of compounds, which resulted in the narrow safety margin. Further modifications of the thiazolo-triazole fragment will be the focus of our next efforts with the aim of reducing the neurotoxicity of these compounds.
